# Sustainable Cold Mix Asphalt Repair: An Analytic Hierarchy Process–Grey Relational Analysis Optimization Framework

**DOI:** 10.3390/ma18102265

**Published:** 2025-05-13

**Authors:** Li Li, Dongwen Guo, Li Teng, Chongmei Peng, Runzhi Yang

**Affiliations:** 1 School of Mechanics and Engineering Science, Shanghai University, Shanghai 200444, China; dongwenguo@shu.edu.cn; 2 Shanghai Urban Operation (Group) Co., Ltd., Shanghai 200023, China

**Keywords:** pothole repair optimization, cold mix asphalt (CMA), highway maintenance, grey relation analysis (GRA)

## Abstract

Cold mix asphalt (CMA) pothole repair is extensively utilized in time-sensitive highway maintenance due to its rapid deployment and all-weather applicability. However, premature failures caused by suboptimal construction practices under operational constraints (e.g., emergency repairs and adverse weather) necessitate frequent reworks, inadvertently escalating material consumption and associated environmental burdens. To address this challenge, this study proposes a quality-driven optimization framework integrating enhanced Analytic Hierarchy Process (AHP) and Grey Relational Analysis (GRA). The methodology systematically evaluates 18 technical parameters across six critical construction phases—grooving/molding, cleaning/drying, bonding layer application, material paving, compaction, and edge trimming—to identify dominant quality determinants. The analysis highlights material placement and compaction as the most significant phases in the repair process, with specific technical parameters such as compaction standardization, paving uniformity, compactor dimension selection, and material application emerging as key quality drivers. To assess the feasibility of the optimized process, a grey relational analysis was adopted to compare the proposed protocol with the cold-patch practices currently adopted by two municipal maintenance agencies in Shanghai, demonstrating superior alignment with an ideal repair benchmark. The developed model empowers highway agencies to achieve dual operational–environmental gains: maintaining urgent repair efficiency while mitigating secondary resource depletion through reduced repetitive interventions.

## 1. Introduction

Asphalt pavement degradation, particularly pothole formation, poses significant challenges to the sustainability of transportation infrastructure. Potholes, primarily induced by heavy traffic loads and exacerbated by water infiltration during winter or rainy seasons [1,2], not only compromise driving comfort but also escalate safety risks, including traffic accidents [3]. Beyond immediate operational hazards, delayed or inadequate repairs contribute to accelerated pavement deterioration, leading to increased lifecycle resource consumption and environmental burdens.

Traditional hot mix asphalt (HMA) has been widely adopted for pothole repairs due to its cost-effectiveness and robust performance under favorable conditions [4]. However, its applicability is severely limited during winter, as heat loss during transportation and placement necessitates excessive energy consumption, exacerbating the environmental footprint of repair operations. Moreover, prolonged traffic closures associated with HMA repairs further contribute to fuel waste and air pollution, underscoring the need for resource-efficient alternatives. In this context, cold mix patching materials (CMPMs) have gained prominence as a sustainable solution for temporary repairs [5].

Composed of mineral aggregates, emulsified or diluted asphalt, and performance-enhancing additives [6], CMPMs eliminate the need for heating, significantly reducing energy consumption and enabling year-round application. Their ease of handling, minimal equipment requirements, and extended shelf life further enhance operational efficiency, particularly in emergency scenarios. Despite these advantages, the expedited nature of emergency repairs often compromises construction quality, leading to premature failures and increased lifecycle resource consumption [7]. This paradox highlights the critical need for scientifically grounded construction protocols to optimize CMPM application, ensuring durable repairs while minimizing material waste and environmental impact.

Despite the growing adoption of cold mix asphalt (CMA) for pothole repairs, the absence of standardized construction protocols tailored to its unique properties remains a critical gap in pavement maintenance practices [8,9,10]. Current repair technologies, primarily designed for hot mix materials, fail to address the specific challenges of CMA application, such as variable weather conditions and time constraints, often resulting in suboptimal performance and increased resource consumption. Therefore, there is an urgent need to evaluate existing pothole repair technologies within the operational constraints of cold mix materials and develop more sustainable and efficient solutions. This study addresses this gap by establishing a comprehensive evaluation framework based on advanced engineering construction methodologies. Various evaluation techniques exist to support such complex decisions, and these can be broadly classified into parametric and non-parametric approaches. Parametric methods rely on assumed functional relationships or predetermined weights in assessing alternatives, whereas non-parametric methods make minimal prior assumptions, often deriving performance metrics directly from observed data [11]. For example, Data Envelopment Analysis (DEA) is a non-parametric technique that evaluates relative efficiency without requiring any preset weights. In contrast, multi-criteria decision-making (MCDM) tools are parametric, as they assign fixed weights to criteria based on expert judgment. Musolino et al. (2017) note that DEA offers an objective efficiency evaluation by optimizing weights for each decision-making unit, whereas MCDM approaches emphasize ranking alternatives according to a weighted set of objectives [12]. The framework developed in this paper aims to optimize cold mix repair processes by enhancing repair performance, minimizing resource consumption, and integrating Multi-Criteria Decision-Making (MCDM) principles to adapt to variable construction conditions in an environmentally responsible manner [13].

MCDM encompasses various methods, including the Analytic Hierarchy Process (AHP) [14], Analytic Network Process (ANP) [15], Simple Additive Weighting (SAW) [16], and TOPSIS [17]. Among these, AHP stands out as a widely adopted and effective tool for practical decision-making due to its structured framework and superior applicability [18,19,20]. For instance, Cao et al. (2019) applied AHP to evaluate asphalt pavement recycling methods, establishing a robust decision-making framework [21]. Similarly, Li et al. (2018) utilized AHP to prioritize pavement maintenance at the network level, quantifying the weights of key factors such as pavement performance, structural strength, traffic load, age, and grade [22]. Han et al. (2022) further extended AHP’s application by developing a quality evaluation model for asphalt pavement construction, segmenting the process into four stages—mixture preparation, transportation, laying, and compaction—and identifying ten critical quality indicators per stage [23].

Despite its strengths, traditional AHP is limited by its reliance on subjective expert judgments, which can introduce bias and uncertainty in weight evaluation [24]. To address this, researchers have proposed modifications to enhance its practicality. For example, Guo et al. (2009) introduced interval numbers to better assess the relative importance of indicators [25], while Wang et al. (2011) replaced the traditional 1–9 scale with a three-point scale to reduce subjectivity in social impact assessments [26]. Ahmed et al. (2017) further mitigated bias by incorporating field data from 28 road sections into pairwise comparisons [27]. Additionally, Luo et al. (2004) demonstrated the applicability of the e^0/4^–e^8/4^ scale for multi-criteria problems, such as evaluating cold patching technologies [28].

To further enhance decision-making robustness, recent studies have integrated Grey System Theory with AHP, leveraging Grey Relational Analysis (GRA) to handle uncertainty and partial data [29]. GRA quantifies relationships between variables under uncertainty, complementing AHP’s structured weighting framework [30,31]. For instance, Yang et al. (2017) combined AHP and GRA to evaluate bridge reinforcement schemes, using AHP to determine indicator weights and GRA to assess relational degrees among alternatives, thereby improving decision outcomes and promoting sustainability [32].

Given its proven effectiveness, the present study develops an AHP–GRA integrated evaluation–optimization framework for cold mix asphalt (CMA) pothole repair. The model systematically covers six construction phases—grooving and molding, pothole cleaning and drying, bonding layer application, material paving, compaction, and edge trimming—and operationalizes 18 technical process indicators that are specifically defined for CMA practice. By fusing subjective expert judgment with data driven grey relational analysis, the framework identifies and ranks the key factors controlling repair quality in each phase, thereby guiding resource efficient, high performance, and environmentally responsible maintenance. This contribution fills the gap between qualitative practice and quantitative efficiency analysis in pavement engineering by providing a structured multi criteria decision-making tool that improves maintenance decision-making under data limited field conditions.

## 2. Quantitative Evaluation Method for Cold Mix Asphalt Pothole Repair Processes

The integration of Analytic Hierarchy Process (AHP) and Grey Relational Analysis (GRA) offers a robust framework for evaluating construction processes under data-limited conditions. AHP, combined with expert scoring, effectively determines the weights of various indicators, making it particularly suitable for assessing complex construction workflows. However, due to its reliance on expert judgments, AHP inherently introduces subjectivity. To address this limitation, GRA is employed as a complementary method, providing a more objective analysis of process performance.

In this study, the AHP–GRA integrated approach is adopted to evaluate and optimize cold mix asphalt pothole repair processes. The methodology involves the following steps: (1) categorizing the repair process based on the properties of cold mix materials; (2) utilizing expert ratings to calculate indicator weights through AHP; (3) establishing a multi-level hierarchical structure for the construction process; and (4) quantitatively evaluating the importance of each indicator using scale scoring methods. Subsequently, GRA is applied to comprehensively compare the preferred construction methods with those currently used by maintenance departments, validating the effectiveness of the optimized approach. This combined framework not only mitigates the subjectivity of AHP but also enhances decision-making accuracy, ensuring the selection of efficient and sustainable repair methods.

### 2.1. AHP Method

Step 1: Hierarchical model construction.

The evaluation model is structured into three hierarchical layers: the goal layer, representing the overall objective of optimizing cold mix asphalt pothole repair processes; the criterion layer, encompassing the key evaluation criteria; and the indicator layer, comprising specific technical parameters that influence repair quality and efficiency.

Step 2: Judgment matrix development.

Pairwise comparisons are conducted among indicators at the same level to construct a judgment matrix using the e^0/4^–e^8/4^ scale method [28]. For a given criterion level *B* with *n* indicators (denoted as *c*_1_, *c*_2_ … *c_n_*), each indicator exerts a varying degree of influence on the criterion. To quantify these influences, weights are assigned to each indicator, reflecting their relative significance. This process results in the construction of n judgment matrices, each representing the pairwise comparisons of factors relative to criterion level *B*.

(1) $$B=\left[\begin{array}{cccc} x_{11} & x_{12} & \ldots & x_{1n}\\ x_{21} & x_{22} & \ldots & x_{2n}\\ \vdots & \vdots & \ddots & \vdots \\ x_{n1} & x_{n2} & \ldots & x_{m} \end{array}\right]\;$$

Step 3: Solving the judgment matrix.

The eigenvalues and eigenvectors of the judgment matrix are calculated using the following equation:

(2) $$B\omega =\lambda_{\max}\omega$$

where $\lambda_{max}$ is the maximum eigenvalue of B; ω is the corresponding eigenvectors of the matrix, which indicate the relative importance of the indicators *c*_1_, *c*_2_ … *c_n_* with respect to B.

Step 4: Hierarchical single ranking and consistency test.

Hierarchical single ranking evaluates the relative importance of indicators at a given level compared to the preceding level. Due to potential inconsistencies in expert scoring, a consistency test is conducted to ensure the reliability of the results. The consistency index (CI) is calculated as follows:

(3) $$\mathrm{CI}=\frac{\lambda_{\max}-n}{n-1}\;$$

(4) $$CR=\frac{CI}{RI}$$

The consistency ratio (*CR*) is calculated to assess whether the judgment matrix meets the consistency condition. If *CR* < 0.1, the ranking is considered consistent and reliable.

Step 5: Hierarchical Total Ranking and Consistency Test.

Table 1 summarizes the hierarchical total-ranking procedure used to derive the global weights for all indicators.

For the overall ranking hierarchy, the consistency index (*CI*) is calculated.

(5) $$\mathrm{CI}=\sum_{i=1}^{n}\;a_{i}\left(\mathrm{CI}\right)$$

Using Equation (4), the consistency ratio (*CR*) is evaluated to determine if the overall ranking satisfies the consistency requirement. *CR* < 0.1 confirms that the overall ranking is consistent and acceptable.

### 2.2. GRA Method

Grey Relational Analysis (GRA) is a robust method for addressing challenges related to information deficiency, model ambiguity, and multiple influencing factors. By quantifying the relationships between variables under uncertainty, GRA enables the evaluation and selection of construction techniques tailored to the unique characteristics of cold mix asphalt repair. This approach facilitates the identification and elimination of low-impact factors, thereby enhancing construction efficiency. The calculation steps for GRA are as follows:

Step 1: Establishing the indicator matrix.

If there are *n* evaluation objects in the quantification evaluation plan, denoted as $D={\left\{D_{1},D_{2}\ldots D_{n}\right\}}^{T}$, and each evaluation object corresponds to m indicators, then we have $D_{i}=\left(d_{i1},d_{i2}\ldots d_{im}\right)$. Therefore, n indicator matrices for comparing and evaluating the evaluation objects can be established as follows:

(6) $$D=\left[\begin{array}{cccc} d_{11} & d_{12} & \ldots & d_{1m}\\ d_{21} & d_{22} & \ldots & d_{2m}\\ \vdots & \vdots & \ddots & \vdots \\ d_{n1} & d_{n2} & \ldots & d_{nn} \end{array}\right]$$

Step 2: Determining the ideal reference.

Assuming the ideal solution is represented by $D_{0}=\left(d_{1}^{0},d_{2}^{0}\ldots d_{m}^{0}\right)$, where $d_{i}^{0}$ represents the optimal value of the i-th indicator, we can establish an evaluation object indicator matrix that includes the ideal solution as follows:

(7) $$\overset{ˉ}{D}=\left[\begin{array}{cccc} d_{1}^{0} & d_{2}^{0} & \ldots & d_{m}^{0}\\ d_{11} & d_{12} & \ldots & d_{1m}\\ d_{21} & d_{22} & \ldots & d_{2m}\\ \vdots & \vdots & \ddots & \vdots \\ d_{n1} & d_{n2} & \ldots & d_{nn} \end{array}\right]$$

Step 3: Standardizing the indicator matrix.

Because the indicators have different units and cannot be directly compared, the matrix must be normalized. Based on the optimal values of the indicators, they are classified into positive and negative indicators. The maximum value among the indicators is chosen as the optimal value for positive indicators, whereas the minimum value is selected for negative indicators. Normalization of positive indicators:

(8) $$S_{ij}=\frac{d_{ij}-d_{ij\left(min\right)}}{d_{ij\left(max\right)}-d_{ij\left(min\right)}}$$

Normalization of negative indicators:

(9) $$S_{ij}=1-\frac{d_{ij}-d_{ij\left(min\right)}}{d_{ij\left(max\right)}-d_{ij\left(min\right)}}$$

After normalizing the indicator matrix, a new matrix is obtained:

(10) $$S=\left[\begin{array}{cccc} 1 & 1 & \ldots & 1\\ S_{11} & S_{12} & \ldots & S_{1m}\\ S_{21} & S_{22} & \ldots & S_{2m}\\ \vdots & \vdots & \ddots & \vdots \\ S_{n1} & S_{n2} & \ldots & S_{nm} \end{array}\right]$$

Step 4: Calculating the correlation coefficient.

The correlation coefficient between the *j*-th indicator in sequence S_i_ and the *j*-th indicator in reference sequence S_0_ is calculated as follows:

(11) $$\xi_{i}\left(j\right)=\frac{min_{i}min_{j}\left|s_{j}^{0}-s_{ij}\right|+\rho min_{i}min_{j}\left|s_{j}^{0}-s_{ij}\right|}{\left|s_{j}^{0}-s_{ij}\right|+\rho min_{i}min_{j}\left|s_{j}^{0}-s_{ij}\right|}$$

The correlation coefficient matrix can be obtained through the calculation in the above equation:

(12) $$E=\left[\begin{array}{cccc} \xi_{1}\left(1\right) & \xi_{1}\left(2\right) & \ldots & \xi_{1}\left(m\right)\\ \xi_{2}\left(1\right) & \xi_{2}\left(2\right) & \ldots & \xi_{2}\left(m\right)\\ \vdots & \vdots & \ddots & \vdots \\ \xi_{n}\left(1\right) & \xi_{n}\left(2\right) & \ldots & \xi_{n}\left(m\right) \end{array}\right]\;$$

Step 5: Computing the degree of association.

(13) $$\gamma_{i}=\frac{1}{n}\sum_{j=1}^{n}\;\xi_{i}\left(j\right)$$

where $\gamma_{i}$ is the degree of association between the *i*-th evaluated object and the ideal reference object increases as the association degree improves, indicating a higher level of alignment with the ideal reference object.

### 2.3. Integrated Evaluation Methods of AHP and GRA

This study will conduct a comprehensive evaluation of the cold patching construction process by integrating AHP and GRA. The integrated evaluation method not only addresses the over-reliance on subjective judgment inherent in AHP but also compensates for the lack of weight consideration in GRA, thereby enhancing the reliability of the evaluation results. The calculation of weight influence correlations in the integrated evaluation method can be modified as follows:

(14) $$\gamma_{i}=\sum_{j=1}^{n}\;\xi_{i}\left(j\right)\times \omega_{j}$$

Based on the initial summary of the cold patching construction process, a comparative analysis questionnaire is designed to solicit expert evaluations. The AHP is employed to calculate and evaluate the weights of various process indicators. A more refined cold patching construction process is proposed based on expert recommendations. Process indicators are quantified, and a corresponding questionnaire is developed to survey the construction processes of various municipal maintenance units. The GRA comprehensive evaluation method is utilized to calculate the correlation between the optimized, actual, and ideal construction process plans to assess the feasibility of the proposed process.

## 3. Quantitative Evaluation Index System for the Pothole Cold Patching Construction Process

**Figure 1:**
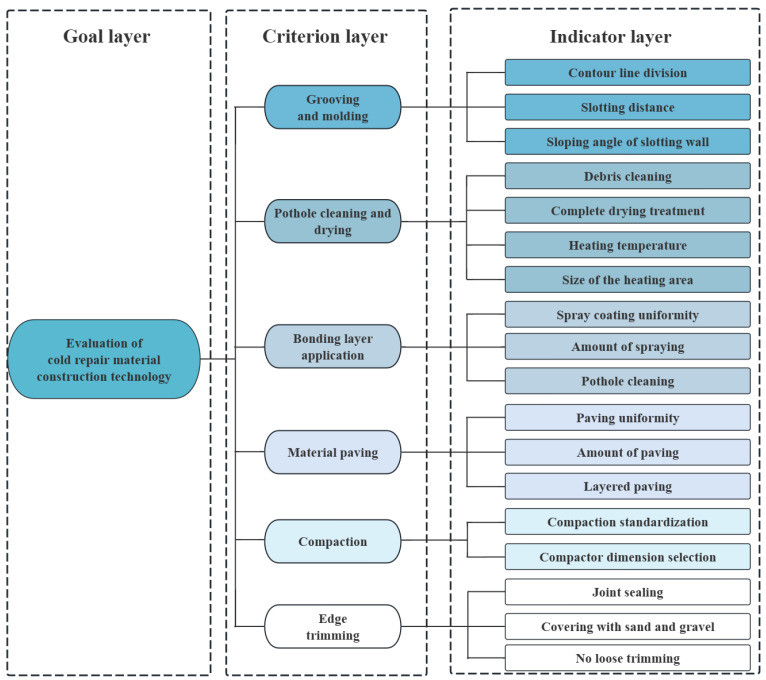
Hierarchy structure diagram of the CMA patching process.

Before applying the AHP–GRA method to evaluate the cold mix asphalt (CMA) pothole repair process, it is essential to establish a robust evaluation index system and select appropriate indicators to ensure the reliability and scientific rigor of the results. Figure 1 illustrates the hierarchical structure linking the goal, criterion, and indicator layers of the CMA pothole repair process. The selection of indicators must adhere to specific principles to guarantee the objectivity and comprehensiveness of the evaluation. In this study, the following principles are followed for indicator selection:

- Systematic principle

The cold mix asphalt pothole repair process comprises a systematic sequence of steps, each contributing differently to the overall repair quality. To ensure comprehensive evaluation, the selection of indicators must encompass all critical steps that influence the final repair outcome. Based on established guidelines, including the Technical Specifications for Maintenance of Highway Asphalt Pavement (JTG 5142) (2019) [33], Technical Specifications for Construction of Highway Asphalt Pavement (JTG F40) (2004) [34], Current and Future Best Practices for Pothole Repair in Illinois (FHWA-ICT-21-003) (2021) [3] and Materials and Procedures for Repair of Potholes in Asphalt-Surfaced Pavements: Manual of Practice (FHWA-RD-99-168) (1999) [35], the primary construction process for cold mix asphalt pothole repair can be summarized into six key steps: (1) grooving and molding, (2) pothole cleaning and drying, (3) bonding layer application, (4) material paving, (5) compaction, and (6) edge trimming.

- Operability principle

The selection of indicators should prioritize simplicity and ease of evaluation by relevant experts. Thus, when selecting appropriate construction process indicators for cold patching materials, existing standards should be integrated with actual construction conditions. Evaluation indicators should be selected with a focus on ensuring the accessibility of the necessary data for evaluation.

- Representativeness principle

The cold patching construction process lacks a well-established standard. Different maintenance units may employ various process steps for repair, some of which may be unrelated to repair effectiveness. Hence, it is crucial to select representative indicators for evaluation.

- Combination of qualitative and quantitative indicators

According to relevant specifications for the pothole repair process, certain processes can be quantitatively represented by specific indicators, such as the amount of repair material used and the drying temperature value. However, some processes cannot be represented by quantitative indicators and must therefore be described qualitatively. Therefore, the principle of combining qualitative and quantitative indicators should be considered in the process of constructing a comprehensive evaluation index system. The quantitative interpretation of every indicator is summarized in Table 2.

## 4. Determine the Weight of Evaluation Indicators for Cold Patching Construction Process

This study involves consulting eight experts with 5–13 years of on-site construction experience to rate the importance of each indicator using a scoring table designed based on the e^0/4^–e^8/4^ scale method. The weights of the indicators are calculated, and the consistency of the calculation results is tested to avoid errors in the expert rating process.

### 4.1. Determine the Weight of Criterion Layer B

The results of the criterion layer calculation are shown in Table 3.

The judgment matrix for the criterion layer in the calculation table shows that λ=6.038, CI = 0.0077, CR = 0.0062 < 0.1. This judgment matrix satisfies the consistency test, ensuring the accuracy of the weights calculated from this matrix. The weights for each expert’s scoring table in the criterion layer are calculated, and the average weight is determined as the final criterion layer weight *ω*_*B*_. The results are shown in Table 4.

Based on the final criterion layer weight results in the table above, the importance order of the criterion layer weights is as follows: Material paving (*B*_4_) > Compaction (*B*_5_) > Bonding layer application (*B*_3_) > Edge trimming (*B*_6_) > Pothole cleaning and drying (*B*_2_) > Grooving and molding (*B*_1_).

### 4.2. Determine the Weight of Indicator Layer C

The calculated results for the weights of the indicator layer under the Grooving and molding (*B*_1_) are shown in Table 5. The calculated judgment matrix in the table yields λ=3.000, CI = 0.0001, CR = 0.0001 < 0.1. This judgment matrix satisfies the consistency test, and the weight calculation is accurate. By following the corresponding calculation steps, the weights for each expert’s scoring table in the indicator layer are calculated.

The average weight is considered as the final weight for the indicator layer under Grooving and molding (*B*_1_), as shown in Table 6.

Based on the results of the indicator layer weights calculation, the importance sequence for each detailed process indicator under grooving and molding is as follows:

Contour line division (*C*_1_) > Sloping angle of slotting wall (*C*_3_) > Slotting distance (*C*_2_).

The final weight b for the remaining indicator layer is listed in Table 7.

### 4.3. Determination of Overall Weightage

From Table 8, we can obtain the weights of 18 specific process indicators relative to the overall objective. By applying Equation (5) to rank the hierarchy consistency, the calculated result is CI = 0.0174, which means CR = 0.0108 < 0.1. The comprehensive weights pass the consistency test.

**Figure 2:**
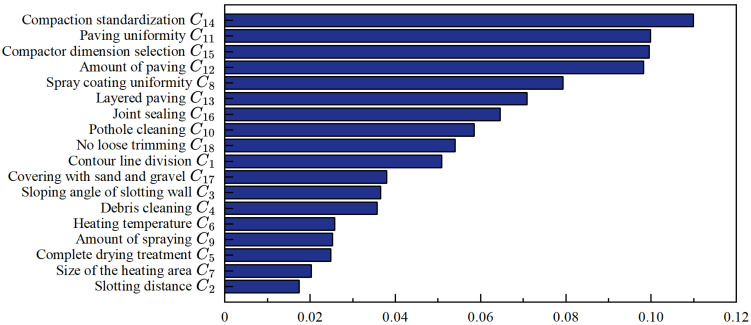
Comparison of comprehensive weights for indicators.

As illustrated in Figure 2, the indicators with the highest comprehensive weights are compaction standardization, compactor dimension selection, material paving quantity, and paving uniformity. These findings underscore the critical importance of standardized paving and compaction in cold mix asphalt (CMA) pothole repair. The quality of these processes directly influences the overall effectiveness and durability of the repair. Given their significant impact, it is recommended that construction personnel prioritize paving and compaction operations on-site. Specifically, attention should be paid to achieving appropriate paving quantity, ensuring uniform material distribution, and achieving thorough compaction to enhance repair performance and longevity.

## 5. Discussions

### 5.1. Comparative Analysis of Cold Patching Construction Technology Options

This study employs a five-level scale (1 = poor, 2 = fair, 3 = average, 4 = good, 5 = excellent) to quantitatively evaluate each indicator within the established evaluation system. The specific scoring criteria for assessing the CMA patching process are detailed in Table 8. Based on the calculated weights and expert opinions, a preliminary patching process for CMA pothole repair is proposed. In practice, emphasis should be placed on critical processes such as material paving and compaction. Under time-constrained or adverse weather conditions (e.g., rainy days), certain steps, such as grooving and pothole drying, may be omitted to enhance operational efficiency without significantly compromising repair quality.

To validate the feasibility of the proposed process, the grey correlation method is applied to compare the recommended process with the actual repair methods used by maintenance agencies in Shanghai. A survey questionnaire, designed based on the evaluation index system, was distributed to two maintenance agencies, yielding 40 and 38 valid responses, respectively. Each process indicator is quantified on a scale of 1 to 5, with the ideal process defined as fully meeting specification requirements and achieving optimal repair outcomes. In the ideal scenario, all process indicators score 5, as summarized in Table 9. This comparative analysis demonstrates the practical applicability of the proposed process, offering a balanced approach to achieving repair efficiency and quality under real-world constraints.

**Figure 3:**
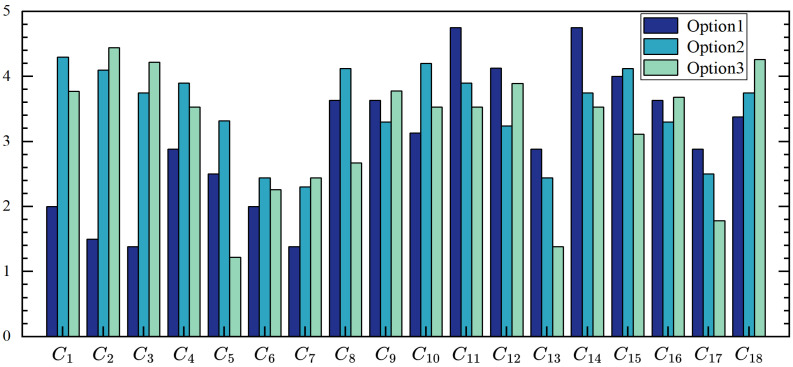
Comparative assessment of scoring indicators for cold patching repair process in pothole.

From Figure 3, it can be observed that the optimized asphalt cold patch construction process focuses more on the material paving and compaction, with a simplified process for initial grooving and cleaning, compared to the two actual maintenance plans.

### 5.2. Calculation Results of Cold Patching Repair Process Scheme for Pothole

After obtaining the statistical results of each option, using the ideal option as the benchmark, GRA correlation coefficients were computed for both optimal and actual construction solutions against the benchmark. A higher correlation indicates that the solution is closer to the ideal one, meaning it can achieve better pothole repair results. The steps are as follows and the calculation results were listed in Table 10.

Step 1: Establishment of indicator matrix

To create a matrix of indicators that includes the ideal solution, refer to Equation (15).

Step 2: Standardization of indicator matrices

At this point, the matrix is not ready for direct calculation; it needs to be normalized. The best value of the indicator for evaluation is 5, which is the maximum value in the rating, then the standardization of the indicator matrix should be standardized using the method of standardization of positive indicators, and the calculation results are shown in Equation (16).

Step 3: Calculate the correlation matrix

Calculate the correlation coefficient of the standardized index matrix. Compute the correlation coefficient matrix of the slot cold patching process indicators according to Equation (11), as shown in Equation (17).

(15) $$\overset{ˉ}{D}=\left[\begin{array}{cccc} 3.77 & 4.30 & 2.00 & 5.00\\ 4.44 & 4.10 & 1.50 & 5.00\\ 4.22 & 3.75 & 1.38 & 5.00\\ 3.53 & 3.90 & 2.88 & 5.00\\ 1.22 & 3.32 & 2.50 & 5.00\\ 2.26 & 2.44 & 2.00 & 5.00\\ 2.44 & 2.30 & 1.38 & 5.00\\ 2.67 & 4.12 & 3.63 & 5.00\\ 3.79 & 3.30 & 3.63 & 5.00\\ 3.53 & 4.20 & 3.13 & 5.00\\ 3.53 & 3.90 & 4.75 & 5.00\\ 3.99 & 3.24 & 4.13 & 5.00\\ 1.38 & 2.44 & 2.88 & 5.00\\ 3.53 & 3.75 & 4.75 & 5.00\\ 3.11 & 4.12 & 4.00 & 5.00\\ 3.69 & 3.30 & 3.63 & 5.00\\ 1.78 & 2.50 & 2.88 & 5.00\\ 4.26 & 3.75 & 3.38 & 5.00 \end{array}\right]$$

(16) $$S=\left[\begin{array}{cccc} 0.72 & 0.87 & 0.22 & 1.00\\ 0.91 & 0.82 & 0.08 & 1.00\\ 0.85 & 0.72 & 0.05 & 1.00\\ 0.65 & 0.76 & 0.47 & 1.00\\ 0.00 & 0.59 & 0.36 & 1.00\\ 0.29 & 0.35 & 0.22 & 1.00\\ 0.35 & 0.31 & 0.05 & 1.00\\ 0.41 & 0.82 & 0.68 & 1.00\\ 0.73 & 0.59 & 0.68 & 1.00\\ 0.65 & 0.84 & 0.54 & 1.00\\ 0.65 & 0.76 & 1.00 & 1.00\\ 0.76 & 0.57 & 0.82 & 1.00\\ 0.05 & 0.35 & 0.47 & 1.00\\ 0.65 & 0.72 & 1.00 & 1.00\\ 0.54 & 0.82 & 0.79 & 1.00\\ 0.70 & 0.59 & 0.68 & 1.00\\ 0.16 & 0.36 & 0.47 & 1.00\\ 0.86 & 0.72 & 0.61 & 1.00 \end{array}\right]$$

(17) $$E=\left[\begin{array}{lll} 0.64 & 0.80 & 0.39\\ 0.85 & 0.73 & 0.35\\ 0.77 & 0.64 & 0.34\\ 0.59 & 0.67 & 0.49\\ 0.33 & 0.55 & 0.44\\ 0.41 & 0.43 & 0.39\\ 0.43 & 0.42 & 0.34\\ 0.46 & 0.74 & 0.61\\ 0.65 & 0.55 & 0.61\\ 0.59 & 0.76 & 0.52\\ 0.59 & 0.67 & 1.00\\ 0.67 & 0.54 & 0.74\\ 0.34 & 0.43 & 0.49\\ 0.59 & 0.64 & 1.00\\ 0.52 & 0.74 & 0.70\\ 0.62 & 0.55 & 0.61\\ 0.37 & 0.44 & 0.49\\ 0.78 & 0.64 & 0.56 \end{array}\right]$$

Step 4: Calculation of comprehensive correlation degree

By incorporating the weights of various process indicators into the calculation of relevance, the results of the comprehensive relevance calculation using the Analytic Hierarchy Process-Grey Relational Analysis method are as follows:

(18) $$R=E\times W=\left(0.5747,0.6024,0.6536\right)$$

where W represents the comprehensive weights of the CMA patching process indicator (Table 7).

Table 10 demonstrates the comparative correlation ranking of the three schemes as γ_1_ > γ_2_ > γ_3_. The optimal option exhibits the highest correlation coefficient, confirming its superior alignment with the ideal benchmark compared to conventional CMA patching practices. The ideal scheme represents the theoretical optimum where all performance indicators achieve peak values, resulting in optimal repair effectiveness. These optimization outcomes offer significant guidance for formulating comprehensive CMA construction specifications. The AHP–GRA framework successfully optimized the repair process, achieving faster re-pair times while maintaining repair quality in emergency situations. While much of the existing literature, such as studies by Cao et al. (2019) [21] and Han et al. (2022) [23], has focused on hot mix asphalt (HMA) repairs, this study presents a framework specifically designed for cold mix asphalt (CMA) repairs, which are typically used for temporary fixes. The AHP–GRA framework in this study addresses the unique challenges of CMA, including performance issues related to emergency repairs and the need for a rapid and effective approach to maintenance in adverse conditions.

## 6. Conclusions

This study establishes a comprehensive evaluation system for cold mix asphalt pothole repair technology, addressing the critical need for effective pavement maintenance. The system, developed based on cold mix material properties and operational requirements, employs an Analytic Hierarchy Process (AHP) to determine process indicator weights. The analysis reveals the following priority order of critical processes: material paving (25.8%), compaction (21.1%), bonding layer application (15.2%), edge trimming (15.0%), pothole cleaning and drying (12.4%), and grooving and molding (10.5%). The evaluation framework incorporates 18 specific process indicators, ensuring comprehensive coverage of all construction aspects and enhancing the system’s scientific rigor. To minimize subjective bias in weight calculations, the AHP methodology integrates index scaling and expert scoring to construct the judgment matrix. Based on this analysis, an optimized construction protocol is proposed, emphasizing compaction standardization, paving uniformity, compactor dimension selection, and amount of paving as primary quality control objectives. The AHP–GRA decision framework significantly contributes to emergency pothole repairs by optimizing the repair process, allowing for faster repair execution without sacrificing the quality of the repair. By prioritizing repairs based on severity and ensuring the most appropriate methods are selected, the framework allows maintenance teams to respond more quickly and effectively in time-sensitive scenarios. This capability is crucial for achieving good repair results under tight time constraints, thus enhancing the overall efficiency of emergency maintenance operations. Validation through an improved AHP-Grey Relational Analysis shows the optimized scheme achieves a correlation coefficient of 0.65, outperforming two conventional field approaches. These results confirm the feasibility and effectiveness of both the evaluation system and the proposed optimization scheme, offering practical solutions for enhancing pothole repair quality and efficiency in real-world maintenance operations.

While this study provides a foundational framework for optimizing cold mix asphalt (CMA) pothole repair processes, future research will focus on validating the model through field trials in different real-world environments. The model’s performance should be assessed under varied climatic conditions, as well as different traffic volumes, traffic types, and repair team compositions to ensure its applicability across a broad range of contexts. Furthermore, expanding the expert panel and integrating additional empirical data will refine the model and improve its robustness. These efforts will enhance the framework’s ability to adapt to diverse operational challenges, ultimately improving the efficiency, sustainability, and cost-effectiveness of pavement maintenance strategies.

## Figures and Tables

**Table 1:** Hierarchical total ranking calculation.

Indicator Layer (C)		Criterion Layer (B)			Hierarchical Total Ordering
	$b_{1}$	$b_{2}$	…	$b_{m}$	
*c*_1_	$c_{1}^{1}$	$c_{1}^{2}$	…	$c_{1}^{m}$	$\overset{m}{\underset{i=1}{\sum }}b_{i}c_{1}^{i}$
*c*_2_	$c_{2}^{1}$	$c_{2}^{2}$	…	$c_{2}^{m}$	$\overset{m}{\underset{i=1}{\sum }}b_{i}c_{2}^{i}$
…	…	…	…	…	…
*c_n_*	$c_{n}^{1}$	$c_{n}^{2}$	…	$c_{n}^{m}$	$\overset{m}{\underset{i=1}{\sum }}b_{i}c_{n}^{i}$

**Table 2:** Quantitative evaluation of the CMA patching process.

Criterion Layer (*B*)	Indicator Layer (*C*)	Interpretation of Indicators
Grooving and molding (*B*_1_)	Contour line division (*C*_1_)	Apply ‘round hole, square patch’ method for durable, uniform repairs.
	Slotting distance (*C*_2_)	Extend repairs 10–15 cm beyond damage for structural integrity and compaction.
	Sloping angle of slotting wall (*C*_3_)	Maintain vertical sidewalls to enhance material bonding and surface contact.
Pothole cleaning and drying (*B*_2_)	Debris cleaning (*C*_4_)	Thoroughly clean the pothole to ensure proper asphalt bonding.
	Complete drying treatment (*C*_5_)	Apply hot air to dry the pothole surface completely before patching.
	Heating temperature (*C*_6_)	Maintain 140–160 °C heating for optimal material fusion.
	Size of the heating area (*C*_7_)	Maintain 10–15 cm heated drying beyond pothole perimeter.
Bonding layer application (*B*_3_)	Spray coating uniformity (*C*_8_)	Apply uniform bonding layer for improved material adhesion.
	Amount of spraying (*C*_9_)	Apply bonding layer at 0.4–0.6 kg/m^2^ to prevent over-saturation.
	Pothole cleaning (*C*_10_)	Prevent dust/debris contamination of bonding layer.
Material paving (*B*_4_)	Paving uniformity (*C*_11_)	Apply uniform continuous paving for proper compaction and strength.
	Amount of paving (*C*_12_)	Overfill center by 3–5 cm for quality repair and material efficiency.
	Layered paving (*C*_13_)	Use layered construction for potholes deeper than 4–6 cm.
Compaction (*B*_5_)	Compaction standardization (*C*_14_)	Compact edges first, then center for optimal density.
	Compactor dimension selection (*C*_15_)	Use slightly smaller compactor for complete material penetration.
Edge trimming (*B*_6_)	Joint sealing (*C*_16_)	Seal joint edges to enhance water resistance and bonding.
	Covering with sand and gravel (*C*_17_)	Cover sealed edges with sand/gravel to enhance skid resistance.
	No loose trimming (*C*_18_)	Ensure tight edge compaction before use.

**Table 3:** Matrix for judging criteria.

Technique for Pit Repair (A)	*B*_1_	*B*_2_	*B*_3_	*B*_4_	*B*_5_	*B*_6_
*B*_1_	1.000	1.000	0.606	0.368	0.472	0.606
*B*_2_	1.000	1.000	0.606	0.368	0.472	0.779
*B*_3_	1.650	1.650	1.000	0.606	0.606	1.284
*B*_4_	2.718	2.718	1.650	1.000	1.284	1.650
*B*_5_	2.117	2.117	1.650	0.779	1.000	1.000
*B*_6_	1.650	1.284	0.779	0.606	1.000	1.000
Weight ω	0.0989	0.1031	0.1631	0.269	0.2095	0.1565

**Table 4:** Weights for judging criteria.

Indicator	Expert 1	Expert 2	Expert 3	Expert 4	Expert 5	Expert 6	Expert 7	Expert 8	$\omega_{B}$
B_1_	0.0989	0.1123	0.1326	0.1092	0.1003	0.1277	0.0626	0.0966	0.1050
B_2_	0.1031	0.0866	0.1622	0.113	0.1045	0.1305	0.1504	0.1456	0.1245
B_3_	0.1631	0.1892	0.0923	0.1649	0.1634	0.1667	0.1505	0.1233	0.1517
B_4_	0.2690	0.2877	0.2534	0.2503	0.2663	0.2204	0.2711	0.2435	0.2577
B_5_	0.2095	0.1765	0.2462	0.203	0.2086	0.1912	0.2277	0.2258	0.2111
B_6_	0.1564	0.1477	0.1133	0.1596	0.1569	0.1635	0.1377	0.1652	0.1500

**Table 5:** Judgment matrix for grooving and molding (*B*_1_).

Grooving and Molding (*B*_1_)	*C*_1_	*C*_2_	*C*_3_
*C*_1_	1.000	2.718	1.650
*C*_2_	0.368	1.000	0.606
*C*_3_	0.606	1.650	1.000
Weight ω	0.5066	0.1863	0.3072

**Table 6:** Weights for grooving and molding (*B*_1_).

Indicator	Expert1	Expert2	Expert3	Expert4	Expert5	Expert6	Expert7	Expert8	$\omega_{c}$
C_1_	0.5066	0.3255	0.5212	0.6088	0.4956	0.5066	0.4286	0.4879	0.4851
C_2_	0.1863	0.1698	0.1495	0.1142	0.1975	0.1863	0.1429	0.1859	0.1666
C_3_	0.3072	0.5047	0.3293	0.277	0.3069	0.3071	0.4285	0.3262	0.3484

**Table 7:** Comprehensive weights for indicators.

**Indicator Layer**	**Grooving and Molding** **(** ***B*** **_1_** **)**	**Pothole Cleaning and Drying** **(** ***B*** **_2_** **)**	**Bonding Layer** **Application** **(** ***B*** **_3_** **)**	**Material** **Paving** **(** ***B*** **_4_** **)**	**Compaction** **(** ***B*** **_5_** **)**	**Edge** **Trimming** **(** ***B*** **_6_** **)**	**Weight Ranking**
	**0.1050**	**0.1245**	**0.1517**	**0.2577**	**0.2111**	**0.1500**	
Contour line division (*C*_1_)	0.4851						0.0509
Slotting distance (*C*_2_)	0.1666						0.0175
Sloping angle of slotting wall (*C*_3_)	0.3484						0.0366
Debris cleaning (*C*_4_)		0.3462					0.0357
Complete drying treatment (*C*_5_)		0.2417					0.0249
Heating temperature (*C*_6_)		0.2506					0.0258
Size of the heating area (*C*_7_)		0.1969					0.0203
Spray coating uniformity (*C*_8_)			0.486				0.0793
Amount of spraying (*C*_9_)			0.1553				0.0253
Pothole cleaning (*C*_10_)			0.3587				0.0585
Paving uniformity (*C*_11_)				0.3714			**0.0999**
Amount of paving (*C*_12_)				0.3649			**0.0982**
Layered paving (*C*_13_)				0.2637			0.0709
Compaction standardization (*C*_14_)					0.5245		**0.1099**
Compactor dimension selection (*C*_15_)					0.4755		**0.0996**
Joint sealing (*C*_16_)						0.4125	0.0646
Covering with sand and gravel (*C*_17_)						0.2425	0.0380
No loose trimming (*C*_18_)						0.3450	0.0540

**Table 8:** Quantitative scoring table for evaluation indicators of cold patching construction technology.

Evaluation Criteria	Scoring Range				
	1	2	3	4	5
Contour line division (*C*_1_)	poor	fair	average	good	excellent
Slotting distance (*C*_2_)	<5 cm	5–10 cm	>20 cm	15–20 cm	10–15 cm
Sloping angle of slotting wall (*C*_3_)	poor	fair	average	good	excellent
Debris cleaning (*C*_4_)	poor	fair	average	good	excellent
Complete drying treatment (*C*_5_)	poor	fair	average	good	excellent
Heating temperature (*C*_6_)	unheated	<70 °C	70–80 °C	80–140 °C	140–160 °C
Size of the heating area (*C*_7_)	<5 cm	5–10 cm	>20 cm	15–20 cm	10–15 cm
Spray coating uniformity (*C*_8_)	poor	fair	average	good	excellent
Amount of spraying (*C*_9_)	>	>	0.2–0.4	0.6–0.8	0.4–0.6
Pothole cleaning (*C*_10_)	$\mathrm{kg}/m^{2}$	$\mathrm{kg}/m^{2}$	$\mathrm{kg}/m^{2}$	$\mathrm{kg}/m^{2}$	$\mathrm{kg}/m^{2}$
Paving uniformity (*C*_11_)	poor	fair	average	good	excellent
Amount of paving (*C*_12_)	poor	fair	average	good	excellent
Layered paving (*C*_13_)	center lower than perimeter	center flush with perimeter	center elevation 1–3 cm	center elevation ≥ 5 cm	center elevation 3–5 cm
Compaction standardization (*C*_14_)	poor	fair	average	good	excellent
Compactor dimension selection (*C*_15_)	poor	fair	average	good	excellent
Joint sealing (*C*_16_)	poor	fair	average	good	excellent
Covering with sand and gravel (*C*_17_)	poor	fair	average	good	excellent
No loose trimming (*C*_18_)	poor	fair	average	good	excellent

**Table 9:** Quantitative scoring analysis of CMA patching options.

Process Indicators	Option 1 (Optimal)	Option 2	Option 3	Ideal Option
Contour line division (*C*_1_)	2.00	4.30	3.77	5.00
Slotting distance (*C*_2_)	1.50	4.10	4.44	5.00
Sloping angle of slotting wall (*C*_3_)	1.38	3.75	4.22	5.00
Debris cleaning (*C*_4_)	2.88	3.90	3.53	5.00
Complete drying treatment (*C*_5_)	2.50	3.32	1.22	5.00
Heating temperature (*C*_6_)	2.00	2.44	2.26	5.00
Size of the heating area (*C*_7_)	1.38	2.30	2.44	5.00
Spray coating uniformity (*C*_8_)	3.63	4.12	2.67	5.00
Amount of spraying (*C*_9_)	3.63	3.30	3.78	5.00
Pothole cleaning (*C*_10_)	3.13	4.20	3.53	5.00
Paving uniformity (*C*_11_)	4.75	3.90	3.53	5.00
Amount of paving (*C*_12_)	4.13	3.24	3.89	5.00
Layered paving (*C*_13_)	2.88	2.44	1.38	5.00
Compaction standardization (*C*_14_)	4.75	3.75	3.53	5.00
Compactor dimension selection (*C*_15_)	4.00	4.12	3.11	5.00
Joint sealing (*C*_16_)	3.63	3.30	3.68	5.00
Covering with sand and gravel (*C*_17_)	2.88	2.50	1.78	5.00
No loose trimming (*C*_18_)	3.38	3.75	4.26	5.00

**Table 10:** Comprehensive correlation degree of cold patching repair process scheme for potholes.

CMA Patching Option s	Option 1 (Optimal)	Option 2	Option 3
Overall correlation γ	0.6536 (γ_1_)	0.6024 (γ_2_)	0.5747 (γ_3_)

## Data Availability

The original contributions presented in this study are included in the article. Further inquiries can be directed to the corresponding author.
